# Anatomical Alterations in Plant Tissues Induced by Plant-Parasitic Nematodes

**DOI:** 10.3389/fpls.2017.01987

**Published:** 2017-11-16

**Authors:** Juan E. Palomares-Rius, Carolina Escobar, Javier Cabrera, Alessio Vovlas, Pablo Castillo

**Affiliations:** ^1^Department of Crop Protection, Institute for Sustainable Agriculture (CSIC), Córdoba, Spain; ^2^Plant Biotechnology and Molecular Biology Group, University of Castilla La Mancha, Toledo, Spain; ^3^A. P. S. Polyxena, Bari, Italy

**Keywords:** *Ditylenchus*, *Heterodera*, giant cell, *Globodera*, *Meloidogyne*, *Rotylenchulus*, syncytium, *Xiphinema*

## Abstract

Plant-parasitic nematodes (PPNs) interact with plants in different ways, for example, through subtle feeding behavior, migrating destructively through infected tissues, or acting as virus-vectors for nepoviruses. They are all obligate biotrophic parasites as they derive their nutrients from living cells which they modify using pharyngeal gland secretions prior to food ingestion. Some of them can also shield themselves against plant defenses to sustain a relatively long lasting interaction while feeding. This paper is centered on cell types or organs that are newly induced in plants during PPN parasitism, including recent approaches to their study based on molecular biology combined with cell biology-histopathology. This issue has already been reviewed extensively for major PPNs (i.e., root-knot or cyst nematodes), but not for other genera (viz. *Nacobbus aberrans, Rotylenchulus* spp.). PPNs have evolved with plants and this co-evolution process has allowed the induction of new types of plant cells necessary for their parasitism. There are four basic types of feeding cells: (i) non-hypertrophied nurse cells; (ii) single giant cells; (iii) syncytia; and (iv) coenocytes. Variations in the structure of these cells within each group are also present between some genera depending on the nematode species viz. *Meloidogyne* or *Rotylenchulus*. This variability of feeding sites may be related in some way to PPN life style (migratory ectoparasites, sedentary ectoparasites, migratory ecto-endoparasites, migratory endoparasites, or sedentary endoparasites). Apart from their co-evolution with plants, the response of plant cells and roots are closely related to feeding behavior, the anatomy of the nematode (mainly stylet size, which could reach different types of cells in the plant), and the secretory fluids produced in the pharyngeal glands. These secretory fluids are injected through the stylet into perforated cells where they modify plant cytoplasm prior to food removal. Some species do not produce specialized feeding sites (viz. *Ditylenchus, Subanguina*), but may develop a specialized modification of the root system (e.g., unspecialized root galls or a profusion of roots). This review introduces new data on cell types and plant organs stimulated by PPNs using sources varying from traditional histopathology to new holistic methodologies.

## Introduction

More than 4,100 species of plant-parasitic nematodes (PPNs) have been identified (Decraemer and Hunt, 2006) and some of them cause damage to economically important crops. A restricted group of genera is considered as major plant-pathogens whereas others are specific to a more limited range of crops. Some estimates suggest that PPNs cause a 77 billion dollar loss in agricultural production worldwide each year (Sasser and Freckman, 1987). Additional losses could be related to food quality and visual imperfections or market devaluation associated with infection symptoms (i.e., carrots or potatoes affected by *Meloidogyne* spp.), restrictions to market exportation due to the imposition of quarantine trade rules, or measures of control aimed at keeping nematodes below damage threshold in the field.

Most nematode damage occurs through direct alteration of plant cells, usually interfering with the normal cell cycle or by withdrawing nutrients from cell cytoplasm. However, some groups also act as virus vectors of nepo- and tobraviruses (Longidorids and Trichodorids, respectively; Decraemer and Robbins, 2007). Furthermore, PPNs could interact with other plant-pathogens to increase damage to the plant or to break plant resistance (i.e., vascular fungal diseases; Back et al., 2002). In addition, some microorganisms pathogenic to grazer animals have been associated with galls produced by anguinid nematodes (McKay and Ophel, 1993). These issues, caused by PPNs, have resulted in quarantine regulations [i.e., ruled by European and Mediterranean Plant Protection Organization (EPPO) and Association of South East Asian Nations (ASEAN)].

The aboveground symptoms of root nematode damage are usually unspecific and associated with nutrient deficiency, incipient wilt, stunting, poor yield, and sometimes plant death. Very few symptoms in plants can be associated unequivocally with PPNs as they are usually difficult to detect, with the exception of galls in roots or stems and necrosis or deformations in some hosts caused by specific species. PPNs can feed on all plant parts, including roots, stems, leaves, flowers, and seeds. For this feeding and interaction with plants, they need a stylet (a hollow mouth spear, like a hypodermic needle), which is highly variable in length and shape. Furthermore, PPNs usually possess three to five pharyngeal glands that produce secretions, most of which are emitted thorough the stylet, that assist plant-nematode interaction (i.e., penetration, internal migration, and parasitism). Other glands (amphids, phasmids, adanal glands, and the excretory/secretory system) as well as hypodermis secretions are important in nematode cross-talk with plants (Rosso et al., 1999; Haegeman et al., 2012). PPNs can be classified as: (i) Ectoparasites: the nematode remains outside of the plant and uses its stylet to feed from the plant root cells; (ii) Semi-endoparasites: nematodes partially penetrate the plant and feed at some point during their life cycle; (iii) Migratory endoparasites: nematodes spend much of their time migrating through root tissues destructively feeding on plant cells; and (iv) Sedentary endoparasites: the nematode spends the majority of their life span sedentary inside the plant tissue establishing a highly specialized parasitism. Groups iii and iv are the most important in terms of crop losses. There are four basic types of feeding cells: (i) non-hypertrophied nurse cells; (ii) single giant cells; (iii) syncytia; and (iv) coenocytes. This variability of feeding sites may be related in some way to PPN life style (migratory ectoparasites, sedentary ectoparasites, migratory ecto-endoparasites, migratory endoparasites, or sedentary endoparasites). Some species do not produce stable feeding sites associated with their parasitism, and in such cases the parasitized cells usually die (i.e., *Trichodorus, Paratrichodorus, Tylenchorhynchus*). Apart from their co-evolution with plants, the response of plant cells and roots are closely related to feeding behavior, the anatomy of the nematode (mainly stylet size, which could reach different types of cells in the plant), and the secretory fluids produced in the pharyngeal glands. These secretory fluids are injected through the stylet into perforated cells where they modify plant cytoplasm prior to food removal. In some cases, the effect of nematode parasitism is not only associated with the feeding site, but it extends to adjacent tissues; for example, in the case of *Meloidogyne* spp. or *Nacobbus aberrans*, the first produce coenocytes and the second a syncytium, both with similar cell proliferation around the feeding sites that finally form a root gall. Nematode mode of interaction with plants is an active field of research targeting the design of effective new control strategies. This review intends to describe those plant-nematode interactions that cause specific alterations in plant cells related to their feeding habit. Because of the extensive range of PPNs, only important genera of major groups with specific effects in plants will be studied.

## Plant morphogenesis induced by nematodes

### Stem, leaf, seed, and root gall nematodes

**Figure 1:**
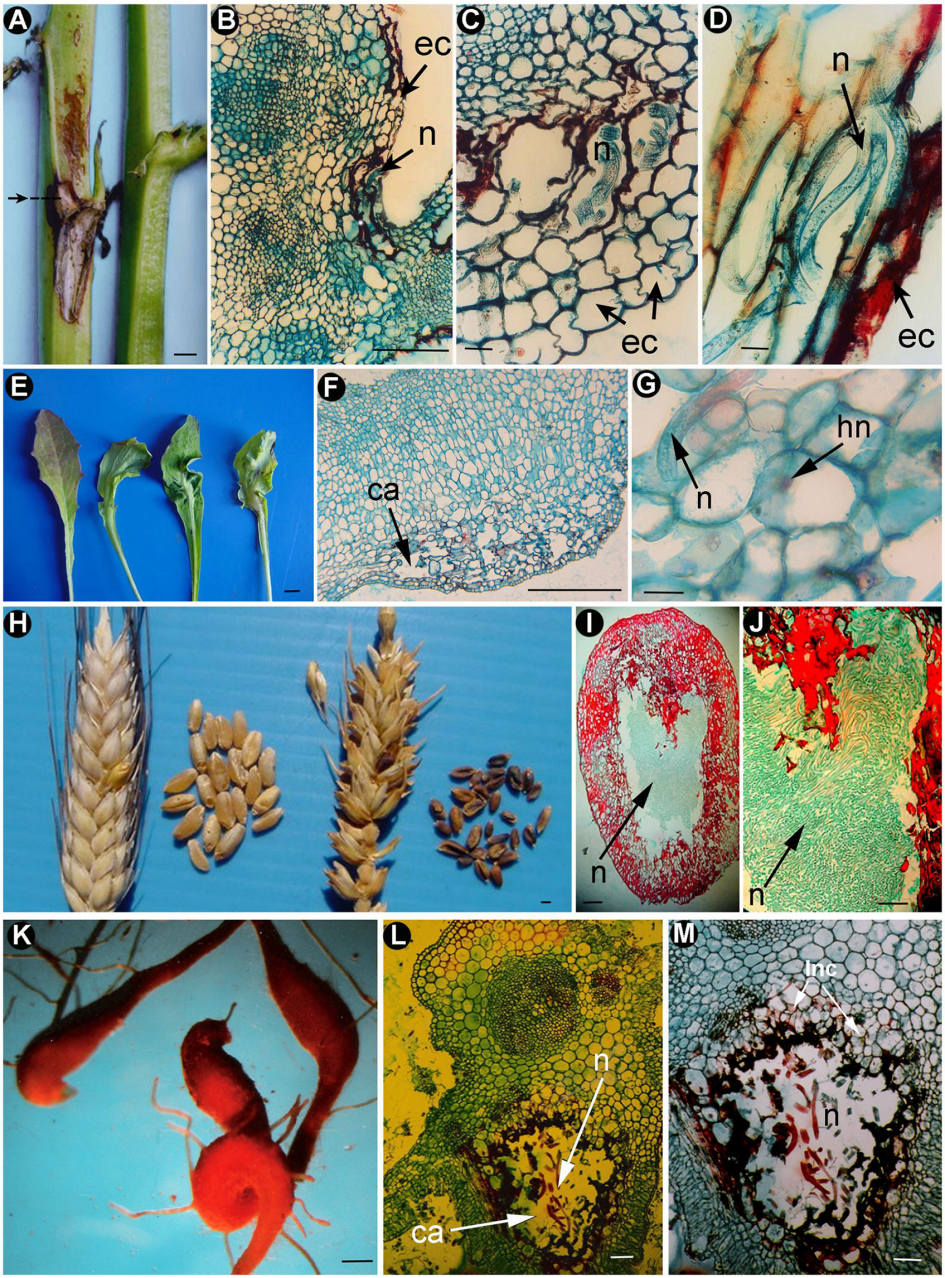
Morphogenesis caused by stem, leaf, seed and root gall nematodes. *Ditylenchus gigas* **(A–D)**: **(A)** Necrotic area on stem (arrowed). **(B–D)** Cross sections of parenchymatic tissues of the stem showing under-epidermic cavities surrounded by necrotic cells and nematode body portions (Vovlas et al., 2011; with permission of John Wiley and Sons). *Ditylenchus oncogenus* **(E–G)**: **(E)** Leaf midrib nematode-induced galls, showing different deformation degrees. **(F)** Longitudinal section of parenchyma of a stem portion showing sub-epidermal cavities (ca) surrounded by necrotic cells. **(G)** Cross-section of flower parenchyma showing a nematode (n), and hypertrophied nuclei (hn) in the attacked cells (Vovlas et al., 2015b; with permission of Cambridge University Press). *Anguina tritici* **(H–J)**: **(H)** Healthy (left) and infected (right) spike and seed galls of wheat. **(I,J)** Cross-sections of wheat-seed showing severe infection induced by the nematode (n) and the high number of nematodes inside the grain (Source: N. Vovlas). Root-galls caused by *Subanguina radicicola* **(K)** on *Poa annua* (Source: N. Vovlas). *Subanguina moxae* **(L,M)**: Cross-sections of foliar galls from *Artemisia* sp. showing cavities (ca), nematodes (n), and a layer of nutritious cells (lnc) (Source: N. Vovlas). ca, cavity; ec, epidermic cell layer; hn, hypertrophied nuclei; lnc, layer of nutritious cells; n, nematode. Scale bars: **A,F,H,K** = 1,000 μm; **B,E** = 500 μm; **C,D,G,J** = 50 μm; **I,L,M** = 100 μm.

These groups of nematodes use films of water to migrate up the plant stem and are therefore more damaging under wet and cold conditions. The fourth-stage juveniles penetrate plant trough buds, petioles, lenticels, or stomata and subsequently move intercellularly through the middle lamella. Symptoms in the plant are leaf or bulb deformities, short internodes, and in some species true neoplastic tissues similar to galls are formed (Figures 1A–G). In most hosts, these nematodes induce extensive cell separation, some necrosis, and hypertrophy (Figures 1B–D,F,G). Usually, several adjacent cells, not directly penetrated by the nematode stylet, exhibit cytological features such as a granulated cytoplasm with hypertrophied nuclei and nucleoli. These cells could be called nurturing cells and they proliferate amongst pith parenchyma and vascular bundles in some plants close to the feeding sites formed by the cavity within the gall (Watson and Shorthouse, 1979; Vovlas et al., 2015a). Some authors as early as Goodey (1935), Krusberg (1961), and Watson and Shorthouse (1979), related this specific plant morphogenesis to the number of meristematic cells, as cortical parenchyma is associated with cell separation only, while meristematic cells are more commonly related with gall formation. The most important species of the genus *Ditylenchus* (“stem nematodes”) is *Ditylenchus dipsaci* because of its wide range of possible hosts and the damage it causes to plants. Other species that cause crop damage include *D. gigas, D. destructor, D. angustus*, and *D. africanus*. However, most species within this genus are fungal-feeders in the soil. Depending on the species, they could infect a broad number of plants (i.e., *Ditylenchus dipsaci*) or be specifically associated with some plants (i.e., *D. oncogenus* to *Sonchus bulbosus* or *D. gigas* to broad beans) (Vovlas et al., 2015b). Plant responses could also differ depending on the nematode-species or their specific host, for example in the species complex group of *D. dipsaci*, some hosts (rye and oats) produce an excessive number of tillers and develop puffy sheaths (Hawn, 1969); in other hosts, leaves produce small pale-green swellings which contain aggregations of nematodes (Campbell and Griffin, 1973), deformed leaves or bulbs as in garlic or onion (Sturhan and Brzeski, 1991), or crown-canker in sugar beet (Castillo et al., 2007). However, some species from this complex group (*Ditylenchus dipsaci* s.l.) are composed of a number of biological races and populations differing in host preferences and occur at a different stage of speciation and reproductive isolation, and probably they could be separated species (Sturhan and Brzeski, 1991). It has been proposed that *D. dipsaci* includes at least seven potential species (Subbotin et al., 2005): *D*. *dipsaci* sensu stricto and six putative species named as *Ditylenchus* sp. B from *Vicia faba* L., *Ditylenchus* sp. C from *Cirsium arvense* (L.) Scop., *Ditylenchus* sp. D from *Pilosella* spp., *Ditylenchus* sp. E from *Crepis praemorsa* (L.) Tausch, *Ditylenchus* sp. F from *Leontodon autumnalis* L., and *Pilosella officinarum* (L.) F.W.Schultz and Sch.Bip. and *Ditylenchus* sp. G from *Plantago maritima* L. Some of these have been recently separated as individual species (i.e., *D. weischeri* or sp. C and *D. gigas* or sp. B; Chizhov et al., 2010; Vovlas et al., 2011).

Seed gall nematodes (*Anguina tritici*) were the first PPNs recorded by Needham (1744) in wheat seed-galls. They have a similar life cycle to *Ditylenchus* species where they infect aerial plant organs, with the exception that they feed ectoparasitically on growing points and leaf bases until they reach the inflorescence in some species (Siddiqi, 2000). They produce galls on flowers, seeds, leaves, and roots (Figures 1H–J). Some species (e.g., *Anguina funesta*) can transport *Clavibacter toxicus* attached to their cuticle to rye grass ears. This bacterium produces toxins that are poisonous to grazing sheep in infested grass in Australia (Bird, 1985). In general, *Anguina* spp. produce a similar morphogenesis in plants to the galls produced by *Ditylenchus* species, with the presence of hypertrophied cells and hyperplasia in gall tissues. However, they seem more specialized in comparison to the galls produced in some hosts by *D. dipsaci* (i.e., garlic or onion). Galls of seed gall nematodes usually develop in place of ovules, less commonly in place of stamens, and rarely on glumes or rachides and are used for nematode survival and dispersal (Figure 1H; Stynes and Bird, 1982). These galls show a considerable thickening of the cell wall of the peripheral outermost layer of cells that probably have a role in the protection of anhydrobiotic second-stage juveniles and gall integrity (Figures 1I,J: Stynes and Bird, 1982; Fattah and Al-Assas, 2010). A cavity is formed by numerous interconnected cells with irregular shape and contains several hypertrophied nuclei with several nucleoli, and a granular cytoplasm (Figures 1I,J). Nematode containing cells are surrounded by a layer of nutritious cells with several hypertrophied nuclei, each containing several nucleoli. These cells could function as a nutrient sink area from the plant (Skinner et al., 1980; Sobczak et al., 1997). The galls of some species, such as *Subanguina picridis* infecting Russian knapweed (*Rhaponticum repens*), have a well-defined zone of numerous nurturing cells and the cells among the nematodes do not become necrotic (Watson, 1986). Plant mechanisms that support parasitism and how those cells act as a nutrient sink tissue for the nematode are important points of study during interaction analysis.

Other species infect aerial plant parts, such as leaves, stems, or inflorescences, and usually they only become established in actively growing undifferentiated tissues. Interestingly, *Anguina* spp., *Paranguina* spp., and *Subanguina* spp. are parasites of monocotyledonous plants with a broad range of hosts that produce one generation per gall with the second-stage juvenile as the invasive stage (Figures 1L,M). *Mesoanguina* shows narrow host specificity, with two morphologically distinct generations per gall and with their third-stage juvenile as the invasive stage. *Heteroanguina* genus parasitize monocots and dicots producing one generation per gall with their fourth-stage juvenile as the invasive stage (Chizhov and Subbotin, 1985). Furthermore, Anguinidae phylogeny obtained using rDNA data shows an evolutionary specialization apparently related to an evolutionary trend in gall development: from abnormal swelling and growth of infested plant organs toward small localized galls, and from infestation of vegetative parts toward generative organs (Subbotin et al., 2004). In addition, there are high levels of co-speciation events between the phylogenies of anguinids parasitizing Poaceae and their host grasses (Subbotin et al., 2004).

These perturbations in leaf tissues are similar to those occurring in compatible interactions with some mites that produce galls. The first reaction of a cell is probably the production of a callous related to the puncturing action of the feeding nematode; this has been documented in gall cells produced by some mites (Stynes and Bird, 1982). Usually the punctured cells die, but in a compatible interaction, the surrounding cells, which become a nourishing tissue, are activated by the continuous feeding of the nematode and cell division occurs. These cells are characterized by a dense cytoplasm, small vacuoles, and enlarged nuclei and nucleoli (Westphal and Manson, 1996) and seem to be activated by the continuous feeding of nematodes as cells are killed by the feeding process and new cells are incorporated into this layer. Recently, the interaction of *Ditylenchus gallaeformans*, which induces galls on the inflorescences of *Miconia albicans* and *Miconia ibaguensis*, has been studied in detail (Ferreira et al., 2017). This interaction showed that instead of flowers, the axes of the galled inflorescences are surrounded by emergences with nutritive tissues lining the larval chambers. The nutritive tissues of these galls have totipotent cells, originating new tissues with dermal, ground, and vascular tissues providing these galls with indeterminate growth. Furthermore, the new development for these types of nematodes, *D. gallaeformans* induces a long-distance impact on fruits, which have an increased number of carpels. Ferreira et al. (2017) suggest that such long-distance effects may compensate for the damage of the galls inducing mechanisms by favoring, at least partially, its host plant fitness.

*Subanguina radicicola* is the only known anguinid that parasitizes roots. It induces galls on the roots of several grasses, barley, and rye and occurs widely in Europe (Siddiqi, 2000). Galls have a small size (less than 5 mm long). They can be found either on the root apex or along the root axis, and sometimes the infected plants have numerous lateral roots (Figure 1K; Vovlas, 1983). In the case of *S. radicicola*, large cavities are formed in the root cortex, but collapsed and enlarged cells are also found in the endodermis, pericycle, and vascular parenchyma. Those changes cause asymmetry of the central cylinder, provoking abnormal functioning of the root and a reduction in plant growth (Vovlas, 1983).

### Root nematodes

#### Plant ectoparasites

Plant ectoparasites comprise a broad range of nematode families. The feeding habit of these nematodes, their secretions, the population densities, the type of cell selected, and the time of interaction within these cells are important factors in the development of different cell and root structures.

**Figure 2:**
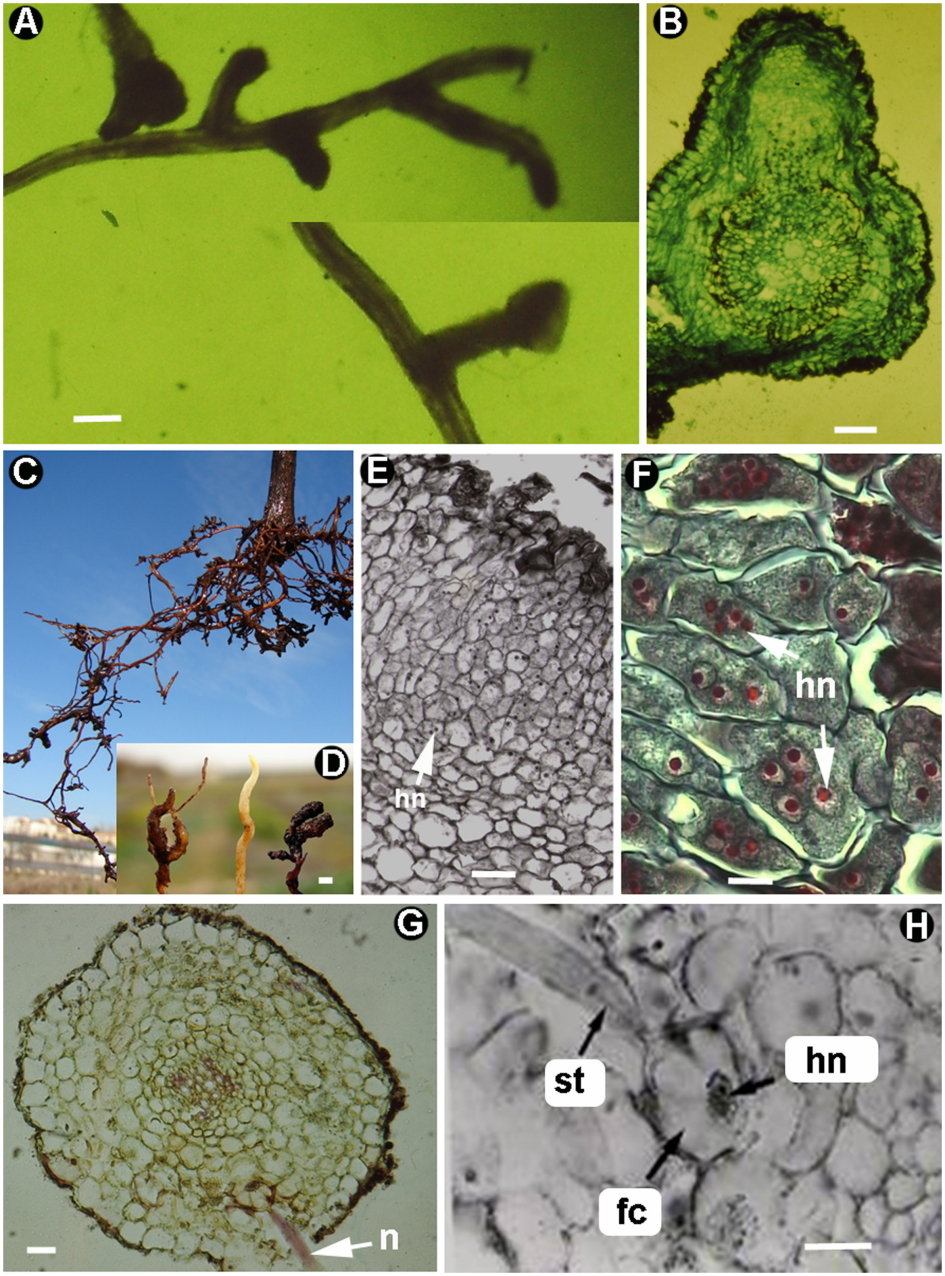
Morphogenesis caused by ectoparasite root nematodes. *Paratrichodorus teres* **(A,B)**: **(A)** Apical-root galls on wheat. **(B)** Cross-section of apical-root wheat gall (Source: N. Vovlas). *Xiphinema index* (C–F): **(C, D)** Apical-root galls in grapevine. **(E,F)** Cross-sections showing multinucleate cells with hypertrophied nucleus (hn) induced by nematode parasitism (Gutiérrez-Gutiérrez et al., 2011; with permission of John Wiley and Sons). *Helicotylenchus oleae* **(G,H)**: Cross-sections of olive roots showing the nematode feeding on a parenchymatic feeding cell (fc) with hypertrophied nucleus (hn) (Source: N. Vovlas). fc, feeding cell; hn, hypertrophied nucleus; n, nematode; st, stylet. Scale bars: **A,D** = 1,000 μm; **B** = 200 μm; **E** = 10 μm; **F,H** = 20 μm; **G** = 100 μm.

Trichodorid nematodes preferably feed on epidermal cells in the elongation regions of rapidly growing roots; they tend to aggregate at the root's apex and stop root growth through gregarious feeding (Wyss, 2010). Usually, they induce abnormal growth of lateral roots and the proliferation of branch roots (Agrios, 2005). Severely infected roots show a smaller root system than non-infected plants, with the presence of fewer roots exhibiting short, stubby, swollen root branches (Figure 2A; Agrios, 2005). Meristematic activity and root growth stop because of the physical feeding effect, and this causes a rounded tip to develop that exhibits differentiation of stellar tissue almost to the apex of the root (Pitcher, 1967); however, cells already formed enlarge abnormally and cause swelling of the root tip (Figure 2B; Agrios, 2005). Other PPNs with similar patterns of ectoparasitic feeding on epidermal cells do not produce the effects caused by Trichodorids, probably because they do not aggregate at specific zones on the root.

Longidorids are large nematodes (up to 12 mm long) that are equipped with long stylets, which allow them to feed deep within plant roots. These nematodes usually feed on root tips, reaching the differentiated vascular cylinder with their long stylets, but they could infect other parts of the root (Figures 2C,D; Cohn, 1970). Two genera have been studied in more detail, *Xiphinema* and *Longidorus*. Both produce galls in the tips by arresting root growth, but with different internal modified cells. *Xiphinema* spp. induce large hypertrophied multinucleated cells (from two to eight nuclei; Figures 2E,F), while *Longidorus* spp. form hypertrophied uninucleate cells (Wyss, 2010). In both cases, these cells are highly active metabolically, with typical hypertrophy, amoeboid-shaped nuclei, increased cytoplasmic density, and abundance of mitochondria, plastids, and rough endoplasmic reticulum (Wyss et al., 1980; Griffiths and Robertson, 1984, 1988). *Xiphinema index* females initially feed in the transition zone between the root apex and cell elongation (Weischer and Wyss, 1980). The odontostyle is inserted through three to four cell layers before feeding starts, and can penetrate to a depth of up to eight cell layers (Weischer and Wyss, 1976). These parasitized cells become necrotic and surrounded by slightly enlarged binucleate cells (Figure 2F: Wyss et al., 1980; Bleve-Zacheo and Zacheo, 1983). The binucleate cells grow to large multinucleate cells by means of synchronous mitoses without cytokinesis and are indispensable for nematode reproduction (Figures 2E,F; Rumpenhorst and Weischer, 1978; Wyss, 1978; Staudt and Weischer, 1992). Once the gall is formed, it becomes attractive to more individuals (Wyss, 2010), probably because of the plant nutrient sink effect. Interestingly, one specific group of species within the genus *Xiphinema* (*X. americanum* group) usually does not induce galls at the tips but instead clusters of short stubby lateral roots are formed (Siddiqi, 1973). In *Longidorus*, a similar process is induced; differing in several points: (i) the feeding habit is at root tips, transforming them into terminal galls (Wyss, 2010); (ii) the initial feeding in one cell also removes the contents of neighboring cells by the production of cell wall holes through dissolution (Robertson et al., 1984); and the initial hypertrophy of individual uninucleate cells is followed by hyperplasia with synchronized cell division and a posterior hypertrophy of these cells (Griffiths and Robertson, 1984). There is still a big question over the putative influence of the different feeding habits in the induction of hypertrophied uninucleate or multinucleated cells, both avoiding cycles of cytokinesis. The feeding habits between *Xiphinema* and *Longidorus* probably differ as *Longidorus* results in a greater uptake of cytoplasm as necrotic cells are frequently found around the odontostyle. Furthermore, the role of effectors could be important in this root interaction, but information on this point is lacking.

Some ecto-endoparasites, such as the genera *Helicotylenchus, Hoplolaimus, Rotylenchus*, and *Scutellonema* could invade roots to feed on cortical or outer stellar cells (Wyss, 2010). Other authors localized parasitized cells adjacent to protoxylem cells (Jones, 1978a) and noted that infection could take place anywhere in the root, with the exception of the root tip (Klinkenberg, 1963). This type of feeding site is beneficial for the female as, after feeding in these modified cells, they lay many eggs (Jones, 1978b). Some species of *Helicotylenchus* could feed in a semiendoparasitic sedentary manner for up to 19 days (Jones, 1978b) even on woody plant roots, for example in the olive (Figures 2G,H; Inserra et al., 1979). The nematode feeds in one cell, which could be enlarged and is surrounded by four or five cells with an enlarged cytoplasmic volume (Jones, 1978a). The food cell is highly active with numerous mitochondria, plastids, amyloplast-like organelles, and rough endoplasmic reticulum (Jones, 1978a). It is also uninucleate and the plasmalema becomes detached from the cell wall in different places, the resultant gap contains vesicles and dense deposits resembling wall fragments. Lipid droplets and proteinaceous deposits have also been documented in food cells (Jones, 1978a). Similarly, *H. oleae* induces a single food cell in the cortex (Figures 2G,H; Inserra et al., 1979). However, *Helicotylenchus microlobus* infecting corn produces a single food cell in the cortex that is the same size as adjacent cortical cells, but with a denser cytoplasm and an enlarged nucleus with a prominent nucleolus (Vovlas and Inserra, 1985). Plant responses in different hosts, the position of parasitized cells in the different plant tissues, and the nematode species could have an important role in the different cell features noted in histological observations.

#### Plant endoparasites

##### Root-knot nematodes (Meloidogyne spp.)

*Meloidogyne* is a genus including more than 90 species. Only a few of them are considered as major pests (*M. incognita, M. javanica, M. arenaria*, and *M. hapla*; Jones et al., 2013). One of their main characteristics is that they are extremely polyphagous (Moens et al., 2009), especially those species with a wide geographical distribution, while others are more specific, for example, *M. baetica* affecting only the wild olive (Castillo et al., 2003a). They often reproduce by mitotic parthenogenesis, with the exception of *M. hapla* or *M. chitwoodi* that reproduce by facultative meiotic parthenogenesis (Berg et al., 2008; Escobar et al., 2015).

**Figure 3:**
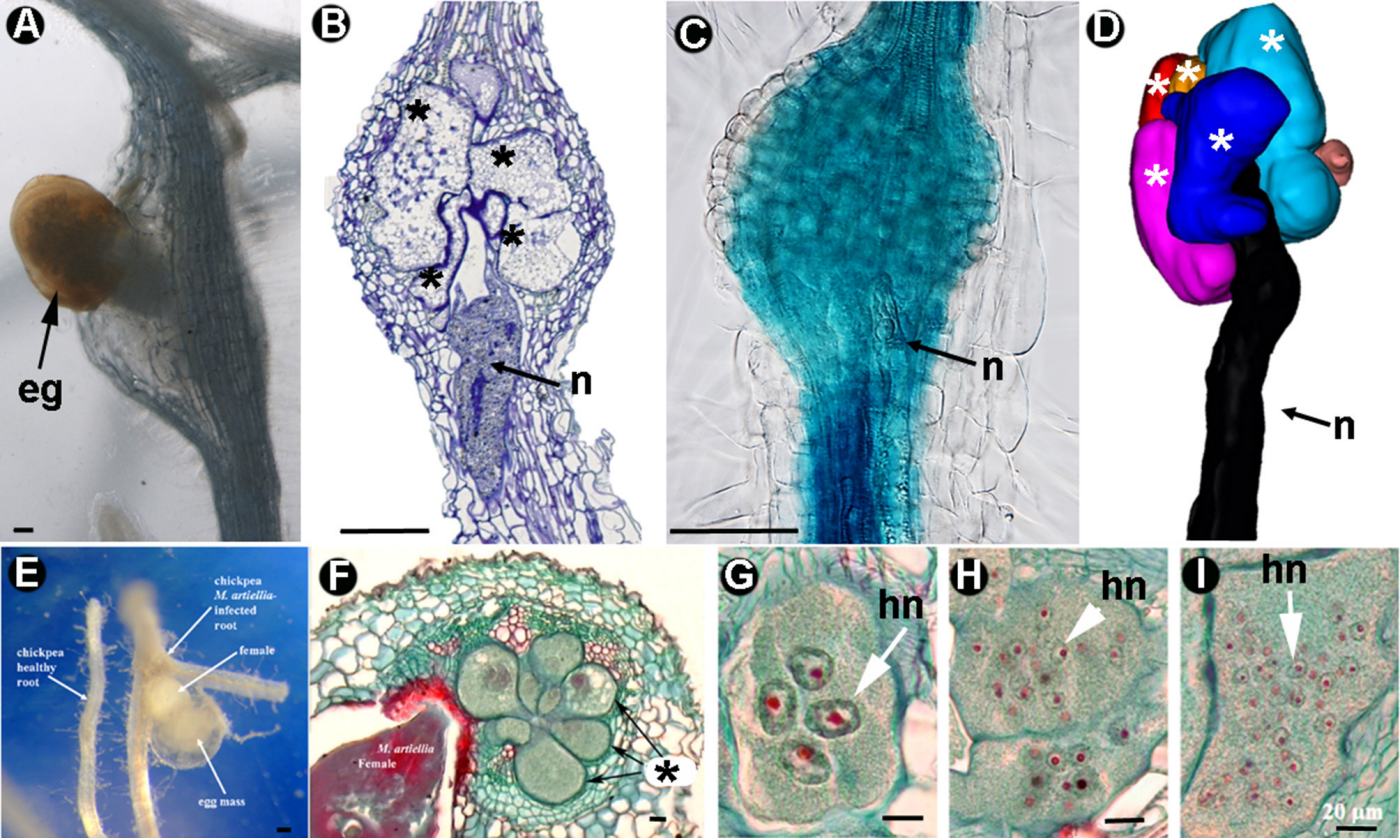
Morphogenesis in root knot nematodes forming galls. *Meloidogyne* spp. **(A–I)**: **(A)** Egg mass (eg) protruding from a gall in a *Cucumis sativus* root infected by *Meloidogyne javanica*. **(B)** Longitudinal section of a gall from *Arabidopsis thaliana* showing multinucleate giant cells (^*^) and anterior region of the nematode (n). **(C)** Vascular tissue of a gall with GUS intense signal from an *Arabidopsis* transgenic marker line. **(D)** 3D reconstruction of giant cells (^*^) from *Arabidopsis thaliana* (Source: C. Escobar). *Meloidogyne artiellia* **(E–I)**: **(E)** Healthy and *M. artiellia*-infected chickpea roots, showing the prominent adult female covered by the egg mass. **(F)** Cross-section of *M. artiellia*-infected root showing the typical feeding site with giant cells (Palomares-Rius et al., 2011; with permission of Elsevier). **(G–I)** Detail of multinucleate giant cells induced by *M. artiellia, M. arenaria*, and *M. javanica* in chickpea roots, respectively (Vovlas et al., 2005; with permission of The American Phytopathological Society). ^*^, multinucleate giant cell; eg, egg-mass; hn, hypertrophied nucleus; n, nematode. Scale bars: **A–C,E** = 100 μm; **F–I** = 20 μm.

Root-knot nematodes (RKN) initiate a subtle interaction with their hosts through intercellular migration after sensing chemical gradients of root diffusates (Teillet et al., 2013). Second-stage juvenile (J2), the infective parasitic form of RKNs, enter the elongation zone of the root and, using cell wall hydrolytic enzymes such as endoglucanases, endoxylanases, pectatelyases, etc., from their subventral glands secreted into the apoplast (Perry and Moens, 2011), they reach the vascular cylinder by entering through the root meristem area. In this way, they considerably reduce mechanical damage to the plant cells as compared to other nematode groups, such as cyst nematodes (CN). Once established, a group of five to eight cells in the vascular cylinder develop into feeding cells, called giant cells (GCs) (Figures 3B,D,F,G,H,I; Escobar et al., 2015). This occurs as a result of refined cross-talk between plant precursor cells and still unclarified nematode effectors (Cabrera et al., 2015a; Truong et al., 2015). The precursor cells of the GCs are not well defined, although pericycle cells are definitely involved in gall/GC development (Cabrera et al., 2014a). Profuse, and mostly asymmetric division of vascular cells, partially resembling the divisions occurring during lateral root formation (Cabrera et al., 2014a), as well as hyperplasia of the surrounding tissues (Escobar et al., 2015), increase root girth through the formation of galls; these are pseudo-organs that function as feeding sites (Figures 3A,E,C). Some *Meloidogyne* species produce small galls (*M. artiellia; M. paranaensis*) (Franklin, 1961; Carneiro et al., 1996) with fewer nuclei but of a larger size, including the nucleoli, for example, *M. artiellia* as compared to *M. arenaria, M. incognita*, and *M. javanica* (Figures 3E–I; Vovlas et al., 2005). Gall size is not phylogenetically related when compared with the latest phylogenetic relationships of the genus (Ali et al., 2015). Perhaps, an important component of these different plant responses is the interaction of plant molecular pathways with the repertoire of effectors produced by the nematode.

Inside the galls, GCs undergo repeated mitosis with partial cytokinesis and endoreduplication or equivalent processes such as defective mitosis or nuclear fusion (de Almeida Engler et al., 2015), leading to DNA amplification, which is thought to be necessary for GC expansion (Escobar et al., 2015). GCs expand, increasing their volume more than 60-fold from 3 days post-infection (dpi) to 40 dpi on average (Cabrera et al., 2015b). Interestingly, the volume of individual GCs does not always correlate with the stage of gall development as GCs probably grow asynchronously. However, the average volume occupied by all GCs as a pool within a gall, show a strong correlation to that of the infection stage (Cabrera et al., 2015b), probably because it is the meaningful functional feeding volume for the nematode. GCs also become transfer cells with cell wall ingrowths and irregular thickenings that increase the effective solute exchange area (Jones and Gunning, 1976; Berg et al., 2008), demonstrating the molecular signatures of this cell type (Cabrera et al., 2014b). Hence, they have irregular shapes, with elongated cell protrusions close to the nematode lip region that can be clearly observed after 3D reconstruction (Figure 3D; Cabrera et al., 2015b; de Almeida-Engler et al., 2016).

After the analysis of single isolated GCs inside the galls, the development of specific techniques combining transcriptomics and cell biology (see cells within the section; Figure 3) identified massive changes in gene expression in *Arabidopsis*, tomato, and *Medicago* GCs ( et al., 2003; Ramsay et al., 2004; Fosu-Nyarko et al., 2009; Barcala et al., 2010; Escobar et al., 2011; Damiani et al., 2012; Ji et al., 2013; Portillo et al., 2013). Thus, huge transcriptional changes encompass GC formation, for example, genes related to secondary metabolism, mostly involved in plant defense are repressed, at least at early-medium stages of infection. However, other stress related genes that are induced, such as those encoding heat-shock proteins, may function as molecular chaperons aiding protein conformation when GC metabolism is actively contributing to nematode feeding (Barcala et al., 2008, 2010). Among the genes with modified expression after nematode infection, a major group are those related to hormone-regulated developmental pathways, particularly those associated to auxin-cytokinin balance, such as *LBD16*, a transcription factor from the lateral organ boundary family crucial for gall/GC and lateral root development (Cabrera et al., 2015b). The irregular shape of GCs (Figures 1C,E) is also accompanied by changes in the cytoskeleton and transcriptional changes in different genes encoding cytoskeletal proteins, such as microtubule-associated (*AtMAP65*), actin depolymerizing factors (*AtADF2*) (Caillaud et al., 2008; Clément et al., 2009), and those from the actin and tubulin family (de Almeida Engler et al., 2004), among others. However, a true dynamic picture of the morphological and molecular changes occurring during GC development is still lacking.

##### Cyst nematodes (Globodera spp. and Heterodera spp.)

**Figure 4:**
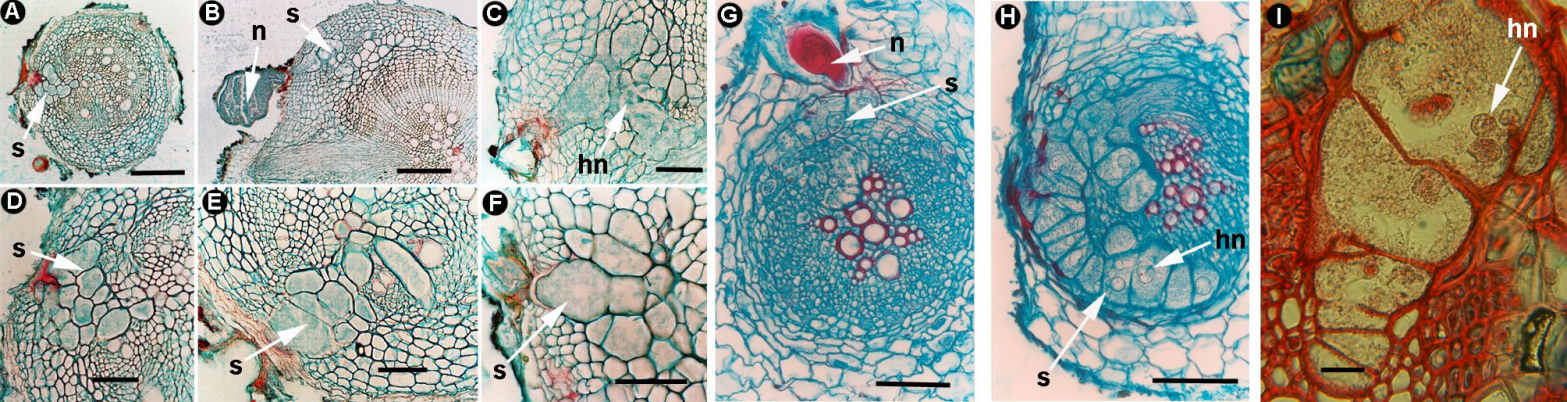
Morphogenesis in cyst nematodes forming syncytia. *Heterodera* spp. **(A–I)**: *Heterodera cruciferae* **(A–F)** Transverse sections of cabbage roots infected by *H. cruciferae* showing the semi-endoparasitic adult female (n) inducing the cortical and stellar syncytium (s) with fused syncytial cells with dense cytoplasm and hypertrophic nuclei (hn) ((Sasanelli et al., 2013); with permission of The American Phytopathological Society). *Heterodera daverti* **(G,H)** Cross-sections of white clover roots showing the semi-endoparasitic adult female (n) inducing the cortical and stellar syncytium (s) with fused syncytial cells presenting dense cytoplasm and hypertrophic nuclei (hn) (Vovlas et al., 2015a; with permission of Springer). *Heterodera filipjevi* **(I)** Detail of syncytial cells in wheat roots showing hypertrophic nuclei (hn) (Source: N. Vovlas). Scale bars: **A** = 1,000 μm; **B,G,H** = 500 μm; **C–F,I** = 100 μm.

Cyst nematodes belong to the subfamily Heteroderinae (Evans and Rowe, 1998). The genus *Globodera* spp. and *Heterodera* spp. contain most of the agronomically important species, although species number is far larger in *Heterodera* (Subbotin et al., 2010) than in *Globodera* (Subbotin et al., 2010). Some of these species could infect woody plants, as is the case for *H. mediterranea* in the olive (Castillo et al., 1999). J2, is the infective stage, in a similar manner to root-knot nematodes, they use their stylet to inject secretions from the gland cells. In contrast, they migrate intracellularly destroying cells from the outer layers of the root due to quick stylet thrusts combined with cell wall degrading and modifying proteins. Their feeding cells are called syncytia, derived from one single cell (initial syncytial cell) that increases its size by fusion of adjacent cells after cell wall dissolution (Figure 4; Sobczak and Golinowski, 2011). In *Arabidopsis*, females of *Heterodera schachtii* usually develop in syncytia from procambial or pericycle cells, whereas males develop in syncytia from pericycle cells (Golinowski et al., 1996; Sobczak et al., 1997). The syncytium can eventually be composed of more than 200 cells when it reaches its maximum size (Figure 4; Hussey and Grundler, 1998). Interestingly, the volume of a syncytium associated with a female can be 10-times larger than that caused by a male, and nuclei enlargement through endoreduplication contributes to this process (Figure 4I; Bohlmann, 2015). These morphological changes are accompanied by profound expression and metabolic changes in syncytia. Accumulation of sugars, starch, and amino acids are common features shared by the GCs of RKNs and syncytia of cyst nematodes (Siddique and Grundler, 2015).

Finally, the juvenile will enlarge and undergo several molts, encompassing the developmental stages J3 and J4, before reaching the adult stage (Figures 4B,G; Barcala et al., 2016). Contrary to most RKN species, they reproduce sexually, the female is fertilized by a free-moving male and deposits the eggs inside its body; this subsequently hardens to provide extra protection for eggs and serves as a resistant cyst (Barcala et al., 2016). Additionally, molecular techniques, such as microaspiration or laser microdissection of syncytia (Anjam et al., 2016) combined with transcriptomic analysis allowed the identification of batteries of genes differentially expressed in soybean roots by *H. glycines* (e.g., Ithal et al., 2007; Klink et al., 2007, 2010). In *Arabidopsis*, expression changes in syncytia indicated a suppression of plant defenses, similar to those that take place in GCs, for example, those genes encoding peroxidases or the induction of genes encoding amino acid transporters (Szakasits et al., 2009).

##### Other plant endoparasites

Nematode-induced feeding cells are derived from different plant tissues depending on the nematode group. In nematodes other than RKNs and cyst nematodes, they can be induced in the cortex or in the vascular cylinder and these may vary from a single feeding cell to several cells forming a feeding site or a syncytium.

**Figure 5:**
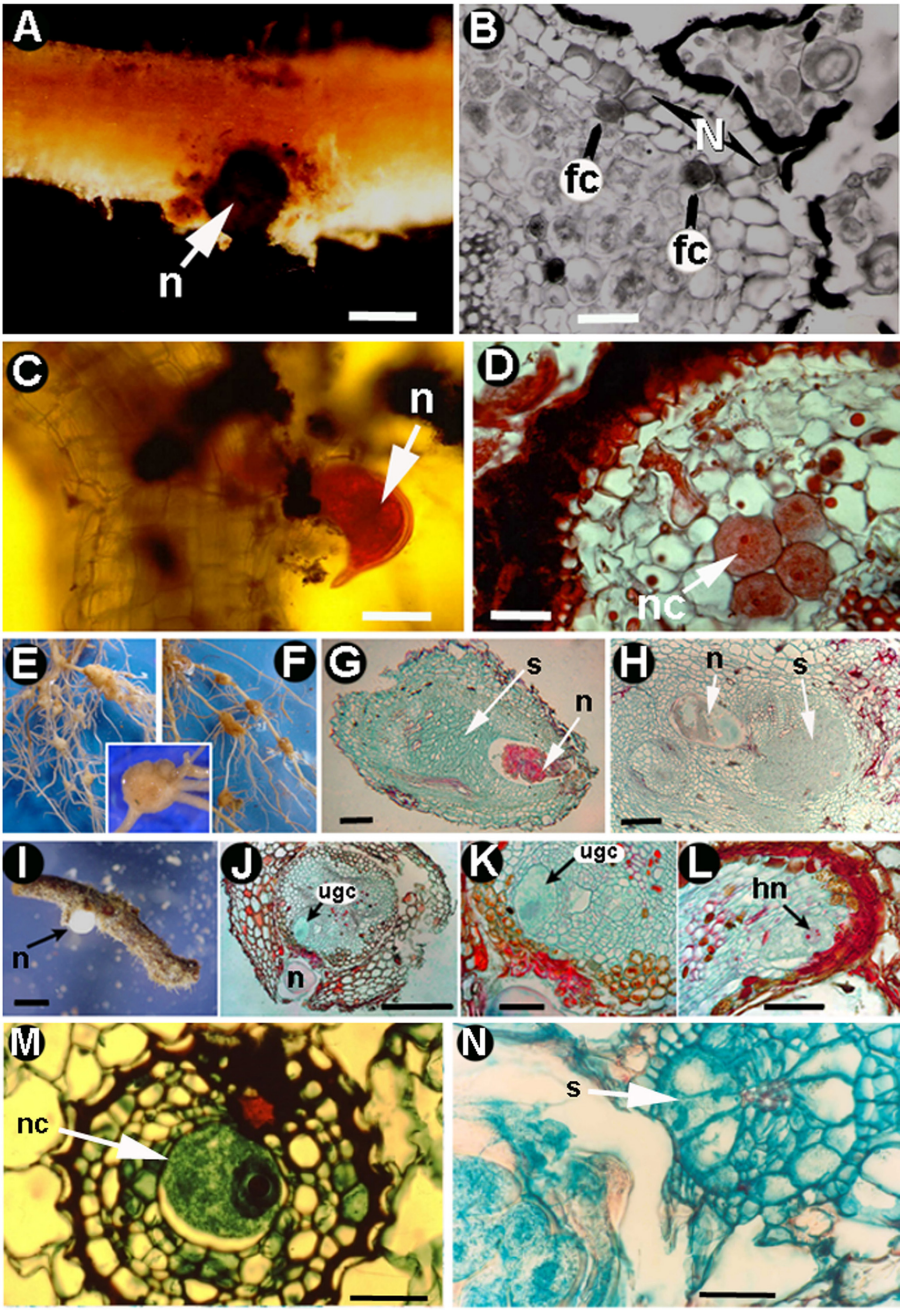
Morphogenesis in root nematodes: other root endoparasites **(A–N)**. *Trophotylenchulus obscurus* **(A,B)**: **(A)** Nematode (n) parasitizing coffee root. **(B)** Cross section of coffee root showing feeding cells (fc) with no evident increase in size and the nematode (Source: N. Vovlas). *Tylenchulus semipenetrans* **(C,D)**: **(C)** Nematode (n) parasitizing citrus root. **(D)** Cross section showing induced nurse cells (nc) with dense cytosols (Source: N. Vovlas). *Nacobbus aberrans* **(E–H)**. **(E,F)** Tomato roots infected by the nematode showing knobs. **(G,H)** Cross sections of tomato roots showing the nematode (n) and the induced syncytium (s) (Vovlas et al., 2007; with permission of Journal of Nematology). *Cryphodera brinkmani* **(I–L)**: **(I)** Root segment of pine with the posterior portion of the body of a white female (n) protruding from the root surface. **(J–L)** Cross sections of pine roots showing nematode female body (n) embedded in the cortical parenchyma and an uninucleate giant cell (ugc) with hypertrophied nucleus (hn) (Vovlas et al., 2013; with permission of Springer). **(M)** Cross section of corn root infected by *Meloidodera charis* showing the single nurse cell (nc) (Source: N. Vovlas). **(N)** Cross section of *Mentha aquatica* root infected by *Meloidoderita kirjanovae* showing the syncytial formation (s) (Vovlas et al., 2006; with permission of Journal of Nematology). Fc, feeding cell; hn, hypertrophied nucleus; n, nematode; nc, nurse cell; s, syncytium; ugc, uninucleate giant cell. Scale bars: **A,I** = 500 μm; **B,G,H,N** = 50 μm; **C,D,J–M** = 100 μm.

*Trophotylenchulus obscurus* parasitizes coffee roots and feeds on a unique cell in the cortex (Figure 5A). This cell has a similar size to neighboring cells, but with a denser cytoplasm and enlarged nucleus with a prominent nucleolus; a large vacuole is formed in senescent nurse cells (Figure 5B; Vovlas, 1987). However, other members of this genus have other feeding habits, for example *T. floridensis* causes the formation of a small number of discrete nurse cells (three to six) in the stellar parenchyma with dense cytoplasm and hypertrophied nuclei and nucleoli in *Pinus clausa* (Cohn and Kaplan, 1983). However, it can also form a syncytium of one to six layers of cortical cells located around the circumference of the root section in a non-cultivated dicot (Inserra et al., 1988). *Tylenchulus semipenetrans* induces a similar feeding site structure to that of *T. floridensis*, with the exception that the cells originate from the cortex and the first feeding cell, into which the lip region of the nematode remains protruded, appears dead and empty with nurse cells distributed around it (Figures 5C,D; Wyss, 2010). Syncytia produced by *Verutus volvingentis* contain dense cytoplasm, enlarged nuclei and nucleoli, and are located within the cortex of the root (Cohn et al., 1984).

*Nacobbus aberrans* induce slight swelling of the root apex which is produced by the migratory feeding habits of juveniles, with a true gall being produced by the adult female (Inserra et al., 1983). The roots show a rosary of bead-like galls, variable in size, with accentuated asymmetry, fragmentation of the stele, hyperplasia of vascular parenchyma, and abnormal proliferation of lateral roots (Figures 5E,F; Inserra et al., 1983; Vovlas et al., 2007). Juveniles move intracellularly, creating necrosis and cavities in the root cortex surrounded by cells containing dense cytoplasm and hypertrophic nuclei; in some hosts, they cause damage to the root stela (Inserra et al., 1983; Vovlas et al., 2007). Inside the gall, females induce a large syncytium derived from incomplete successive fusions of adjacent cells (Figures 5G,H; Vovlas et al., 2007). Cells maintain their individuality in the syncytium, which may involve over 185 cells with dense cytoplasm and hypertrophied nuclei and nucleoli (Figures 5G,H; Vovlas et al., 2007). Some studies reveal that *N. aberrans* s.l. is a complex of species with important differences in host preference (Lax et al., 2014).

Another genus, related to *Heterodera* and *Globodera*, is the cystoid nematode *Meloidodera*, which exhibits some characteristics of *Heterodera* and *Meloidogyne* in its life cycle. The female of these nematodes does not turn into a cyst and its eggs are deposited in a small gelatinous matrix or sometimes retained inside the body. These nematodes induce a GC in the pericycle with a unique irregular hypertrophied nucleus and a variable number of nucleoli; hyperplasia takes place in cells adjacent to the GC (Mundo-Ocampo and Baldwin, 1983a). This GC grows inside the vascular cylinder and therefore has direct contact with the vascular system; plasmodesmata are also concentrated in pit fields, which occur in the thin part of the GC wall adjacent to the vascular parenchyma (Figure 5M; Mundo-Ocampo and Baldwin, 1983a). GCs induced by *Meloidodera* spp. vary in shape and size depending on the nematode and host species as well as stage of root development at the time of infection (Mundo-Ocampo and Baldwin, 1983a). Other Heteroderidae, such as *Cryphodera* and *Sarisodera*, produce GCs in a similar way to *Meloidodera* (Figures 5I–L; Mundo-Ocampo and Baldwin, 1983b, 1984) however, *Atalodera* (Ataloderinae) or the cystoid nematode *Meloidoderita* induces a syncytium (Figure 5N; Mundo-Ocampo and Baldwin, 1983c; Vovlas et al., 2006). These different nematode genera produce variable interactions with plants and have scarcely been studied because of their minor importance as crop pests; however, they are of importance in phylogenic analysis.

In reniform *Rotylenchulus* species, only immature females invade the roots. Two types of plant reaction are induced by these nematodes: (i) a uninucleate GC (*R. macrodoratus*), and (ii) a syncytium (*R. borealis, R. macrosoma, R. parvus*, and *R. reniformis*), both originate from the endodermis (Vovlas et al., 1985). The syncytium is confined to the pericycle layer of the root with most of the cells retaining their individuality and effectively separated from the surrounding cells by a thick cell wall. However, this response seems to be mediated by characteristics of the host or root, because uninucleate GCs originating from a cortical cell and extending from the cortex into the stele have also been observed in thick roots infected by *R. macrodoratus* or in different host species by *R. borealis* (Inserra and Vovlas, 1980; Vovlas et al., 1985). Nevertheless, in wild and cultivated olives, both types of cell response can be induced by *R. macrodoratus* and *R. macrosoma* (Castillo et al., 2003b; Van Den Berg et al., 2016). *Rotylenchus reniformis* could also parasitize cells in the cortex, through formation of a connection to the stele, similar to *R. macrodoratus* or *R. borealis*, but this does not appear to be associated with root diameter. Instead, there appears to be an area of the root with multiple infections that might respond differently to the rest of the root (Razak and Evans, 1976). The cytoplasm of the feeding cells is dense and granular and surrounds a larger and irregularly shaped nucleus with a large nucleolus (Razak and Evans, 1976; Vovlas et al., 1985).

## Molecular interaction between nematodes and plant effectors

Hogenhout et al. (2009) defined an effector as “all pathogen proteins and small molecules that alter host-cell structure and function.” Nematodes secrete effectors to develop their feeding cells, to easily up-take the contents of the cytoplasm, and to move through plant tissues. Those with a more intimate relationship with plants (parasitizing them, but not killing them), as is the case for RKN and CN, change plant developmental processes (mainly by altering the plant phytohormone balance, and the plant cell wall architecture) and also modulate host stress and defense responses [regulation of reactive oxygen species (ROS)] (Lin et al., 2016). They normally use nuclear-targeted, apoplastic, and cytoplasmic effectors (Gardner et al., 2015). Haegeman et al. (2012) noted that “the different lifestyles of PPNs are expected to be reflected in their secretions, which presumably contain effectors with different functions according to the nematode's specific needs.” However, with the exception of a small number of species where the genome or transcriptome has been sequenced, the remaining PPNs are practically unexplored. Another point is the important percentage of nematode-derived sequences without homologies (pioneer genes) in the databases that need to be characterized. Effectors are usually secreted from the pharyngeal glands, the hypodermis, and the amphids. Some of the effectors found in PPNs have been incorporated in genomes by horizontal gene transfer from other microorganisms such as bacteria and fungi (Haegeman et al., 2011a). In this review, we have concentrated on effectors involved in cellular and tissue modification in plants. Other extensive reviews in this subject can be found elsewhere (Haegeman et al., 2012; Favery et al., 2016; Rehman et al., 2016). Some effectors are involved in the invasion, migration, and degradation of host tissues. This has deep implications on the damage provoked to root cells during the nematode migration in endoparasites. Cellulases, pectate lyases, polygalacturonases, xylanases, arabinogalactan endo-1,4-betagalactosidases, and arabinases have been found in many different species of PPNs (Haegeman et al., 2012). Other effectors could help in the action of these enzymes, such as expansins, and they have also been found in a broad range of nematodes (Haegeman et al., 2012; Nyaku et al., 2014). Many of these genes are widely present in PPNs and have been introduced into nematode genomes by horizontal gene transfer from other microorganisms (Haegeman et al., 2011a).

Two different ontogenies of nematode-induced structures are considered as indicated above: the cell which nourishes the nematode and the gall. Gall formation in some cases (such as in RKN) has similarities with nodules induced by endosymbiotic bacteria. For example, PHANTASTICA and KNOX transcription factors, the early nodulin gene ENOD40, and the cell cycle control gene CCS52a are induced in plants during formation of both nodules and GCs (Favery et al., 2002). Genes encoded by RKN, similar to *NodL*, could generate active Nod factors in nematodes (Haegeman et al., 2012) and be involved in nodulation. However, these genes have not been found in the transcriptome of *D. africanus* and *D. destructor*, in which different mechanisms could be used to generate the feeding cells (Haegeman et al., 2009). In this respect, RKN and CN species secrete chorismate mutase (CM; Doyle and Lambert, 2003); this enzyme is proposed to deplete levels of the chorismate precursor, leading to auxin production. There are many nematodes with different feeding sites employing this enzyme (RKN and CN) or even migratory endoparasites that do not produce any specific feeding sites (Haegeman et al., 2011b). In this sense, many differentially expressed genes or the activation of promoters related to auxin (e.g., Cabrera et al., 2014b; Olmo et al., 2017) and ethylene signaling have been observed during the plant-nematode interaction [and in a lesser extent related to giberelic acid (GA), cytokinins, and abcisic acid (ABA)] (Gheysen and Mitchum, 2009; Li et al., 2009; Goverse and Bird, 2011). Moreover, auxins and cytokinins have been found in *H. schachtii* and *M. javanica* secretions by using mass spectrometric analysis (De Meutter et al., 2003, 2005). Furthermore, the role of alterations in the plant hormonal regulation during the feeding site formation became more complex since the discovery in the RKN and CN secretions of proteins that can mimic plant peptide-hormones. One of them is the CLAVATA-like elements (CLE), described initially as plant factors promoting cell differentiation in root and shoot apical meristems (class A; Whitford et al., 2008). Several of these peptides have been found in nematode secretions and in their genomes (RKN and CN; Gao et al., 2001; Abad et al., 2008; Opperman et al., 2008). Other plant peptide hormones, such as C-terminally Encoded Peptide (CEP)-like sequences, are present in *M. incognita* and *M. hapla* but are absent in cyst nematodes (Goverse and Bird, 2011). However, their specific role in the formation of the feeding site is not clearly demonstrated. A multigene phylogenetic analysis of *N. aberrans* with respect to PPNs of all groups confirms its proximity to both CN and RKN (Eves-Van Den Akker et al., 2014). Interestingly, three CLE-like peptides have been identified in the *N. aberrans* transcriptome, two of them contain putative signal peptides and were significantly up-regulated during the sedentary biotrophic phase. However, no CEP-like peptides were identified in the *N. aberrans* transcriptome (Eves-Van Den Akker et al., 2014). In this sense, the unique features of CEP-like peptides in *Rotylenchus reniformis* (syncytium forming nematodes) expand the importance of these effectors in: (i) increasing host nitrate uptake, whilst (ii) limiting the size of the syncytial feeding site produced. However, these CEP domains evolved *de novo* in *R. reniformis* (Eves-Van Den Akker et al., 2016). The presence of CLE genes with greater expression in sedentary phases of *R. reniformis* have also been studied (Wubben et al., 2015). Recently, the genome of *R. reniformis* has been sequenced and more important data linking groups of sedentary nematodes and effectors could be explored in the future (Nyaku et al., 2014). Some effectors have been found in many nematodes, such as the transthyretin-like proteins (TTL; Gao et al., 2003; Furlanetto et al., 2005; Jacob et al., 2007; Bellafiore et al., 2008; Jones et al., 2009; Haegeman et al., 2012), which could target the brassinosteroid signaling pathway, a main mechanism for hormonal regulation in plants that greatly impact plant development (Wei and Li, 2016), but this remains to be proven (Haegeman et al., 2012).

It is established that all PPNs inject effectors before up-take of cytoplasm. Even nematodes that undergo a short feeding process with the plant, create modifications of the cytoplasm and nuclei (i.e., Trichodorids, migratory ectoparasites, or migratory endoparasites; Wyss, 2010). Their mode of action could be to manipulate host transcription as an important strategy for counteracting plant defense responses (Jaouannet and Rosso, 2013; Quentin et al., 2013). However, many aspects of the plant-nematode interaction mediated by nematode effectors that interfere with the plant/cell development remains to be elucidated.

## Conclusions

This review highlights the diversity of plant morphogenesis induced by PPNs. Their complexity increases with an increase in PPN sedentary nature and therefore their requirement for a sustained food supply for nourishment to complete their biological cycle. Interestingly, such PPNs are the most successful parasites and produce relevant economic losses affecting different crops (i.e., RKNs and CNs). This fact has biased the profuse knowledge of PPN interaction to be mainly centered on two genera (*Meloidogyne* and *Globodera*). However, a general trend that takes place in most plant-nematode interactions is the induction and development of feeding cells that exhibit a dense cytoplasm along with nuclear alterations such as a large nucleolus. These findings suggest that nuclear changes must be crucial to sustain nematode feeding, together with a dense cytosol, which is a sign of high metabolic activity. Yet, the size of feeding cells seems more variable, for example the GCs of RKNs can increase to more than 60-fold their volume, whereas *Trophotylenchulus obscurus* induces a single cell with no prominent size increment in comparison to surrounding cells.

In this review, we have described a scenario where a plethora of some interesting plant-nematode interactions in nature have not yet been clearly or deeply studied, they are mere histological descriptions with scarce or no molecular studies of the affected tissues. This makes impossible to compare mechanisms of feeding cell induction or the processes contributing to their maintenance within the plant and therefore it is not currently possible to establish a general and clear picture of commonalities between different feeding sites formed by different PPNs.

## Author contributions

JP-R, CE, JC, AV, and PC conceived the topic. JP-R, CE, JC, AV, and PC wrote the manuscript. JP-R, CE, JC, AV, and PC revised several versions of the manuscript.

### Conflict of interest statement

The authors declare that the research was conducted in the absence of any commercial or financial relationships that could be construed as a potential conflict of interest.
